# PGAP3 is expressed at increased levels in asthmatic ASM and is associated with increased ASM proliferation, contractility and expression of GATA3 and ALOX5

**DOI:** 10.1371/journal.pone.0320427

**Published:** 2025-03-25

**Authors:** Eric Leslie, Marina Miller, Allison Lafuze, Sofya Svyatskaya, Gil-Soon Choi, Joshua L. Kennedy, Yung-An Huang, Taylor A. Doherty, David H. Broide

**Affiliations:** 1 Division of Allergy and Immunology, Department of Medicine, University of California, San Diego, California, United States of America; 2 Department of Internal Medicine, Kosin University College of Medicine, Busan, Republic of Korea; 3 Arkansas Children’s Research Institute, Little Rock, Arkansas, United States of America; University of Mississippi, UNITED STATES OF AMERICA

## Abstract

Post-GPI Attachment to Proteins phospholipase 3 (PGAP3) is a glycosylphosphatidylinositol (GPI) anchor-remodeling gene found on chromosome 17q12-21, which is a locus highly linked to asthma. Genetic association studies have linked PGAP3 SNPs to increased PGAP3 expression as well as asthma exacerbations, severity, and susceptibility. This study compared the levels of PGAP3 mRNA expression quantitated by RT-qPCR in human bronchial airway smooth muscle cells derived from postmortem lungs of asthmatics (ASM-A) to that derived from control non-asthmatics (ASM-NA). ASM-A expressed significantly higher levels of PGAP3 mRNA compared to ASM-NA. As ASM-A expressed higher levels of PGAP3 mRNA we performed functional studies of ASM-NA transfected with PGAP3 to determine if increased PGAP3 expression in ASM influenced ASM function including proliferation and contractility. Functional studies of ASM transfected with PGAP3 demonstrated that increased PGAP3 expression in ASM resulted in increased ASM proliferation and contractility. RNA-seq studies of ASM transfected with PGAP3 demonstrated significantly increased levels of genes linked to asthma including GATA3 and ALOX5. Fifteen genes upregulated by PGAP3 in ASM-NA were detected in asthmatic ASM data sets, underscoring the ability of PGAP3 to induce genes of importance to asthma in ASM. In summary, this study made the novel observation that ASM derived from the lungs of asthmatics express higher levels of PGAP3 compared to non-asthmatics. In addition, when ASM from non-asthmatics are transfected with PGAP3, the increased levels of PGAP3 increase ASM proliferation and contractility, and increase levels of genes previously linked to asthma including GATA3 and ALOX5. Overall, these studies suggest that increased PGAP3 expression in ASM plays a functional role in contributing to the pathogenesis of asthma.

## Introduction

Post-GPI Attachment to Proteins phospholipase 3 (PGAP3) is a gene found on the proximal region of chr:17q12-21, a locus highly linked to asthma [1–3]. Other genes within this locus, including ORMDL3 and GSDMB, have studies not only demonstrating their genetic epidemiologic linkage to asthma, but also studies of their biology implicating them in the pathogenesis of asthma and airway remodeling [2–5]. Several studies of PGAP3 have shown that the PGAP3 SNPs rs2517954, rs2517955, and rs2941504 [6–11] increase asthma exacerbations, severity, and susceptibility [6–8]. All three of these SNPs (rs2517954, rs2517955, and rs2941504) are also associated with increased PGAP3 expression [10,11]. At present little is known about whether lung cells in asthma express PGAP3 at higher levels than non-asthmatics. Previous studies have shown that levels of PGAP3 mRNA are comparable to levels of expression to other 17q12-21 genes (e.g., GSDMB and ORMDL3) in normal human bronchial epithelial cells (NHBE), lung CD4 + tissue-resident memory cells, and peripheral blood leukocytes, but that PGAP3 has very low levels of expression in normal human airway smooth muscle cells [2]. Thus, it is not known whether asthmatic ASM (a key cell in the airway in asthma regulating bronchoconstriction, airway hyperreactivity, and airway remodeling) express PGAP3, as well as which biological pathways regulated by PGAP3 may contribute to the pathogenesis of asthma which is the focus of this study.

PGAP3 (also known as PER1 [12] and PERLD1 [9]) encodes a glycosylphosphatidylinositol (GPI)-specific phospholipase that remodels GPI-anchored proteins (GPI-APs). While PGAP3 has also been found in the cytosol and plasma membrane (https://www.proteinatlas.org/ENSG00000161395-PGAP3) [13], PGAP3’s primary known function is in the Golgi Apparatus to remove unsaturated fatty acids on GPI-APs [12]. This process is also known as GPI-anchor remodeling and is essential for GPI-AP association to lipid rafts within the plasma membrane [12,13]. Since GPI-APs include cell adhesion molecules, enzymes, and receptors [12], an increase in PGAP3 expression related to asthma may impact their function.

We have previously demonstrated that inducing increased PGAP3 expression in normal human bronchial epithelial cells through transfection of a PGAP3 plasmid significantly upregulated the expression of several genes associated with the innate immune response and viral signatures of respiratory viruses associated with asthma exacerbations [14]. Two of the highest expressed genes induced by PGAP3 in NHBE are RSAD2 and OASL, which are anti-viral genes associated with asthma [14]. PGAP3 also upregulated the antiviral gene BST2, which is a GPI-anchored protein [14]. Thus, PGAP3 expression in NHBE regulates expression of genes known to be linked to asthma, and also regulates the bronchial epithelial expression of genes pertinent to the pathogenesis of respiratory viral triggered asthma exacerbations [14].

Airway smooth muscle (ASM) cells from asthmatics are another structural cell in the airway that plays an important role in the pathogenesis of asthma by contributing to several features of asthma including bronchoconstriction, airway hyperreactivity, and airway remodeling [15]. As levels of PGAP3 in ASM from asthma are unknown, in this study we investigated whether levels of PGAP3 were increased in ASM from asthmatics as compared to non-asthmatics. In addition, we performed RNA-seq, bioinformatic, and functional studies in ASM transfected with PGAP3 to determine what genes and pathways were activated by increased PGAP3 in ASM and whether this had functional consequences for ASM in terms of proliferation, and contractility.

## Methods

### Ethics statement

The need for consent was waived by the ethics committee. The investigators using postmortem lung samples had access to specimens and data that were fully anonymized by the time the study was performed. The anonymized archived samples and data were accessed for research purposes from September 1, 2018 to the date of submission of the Research Letter on October 21, 2024.

### Postmortem lungs from asthmatics and non-asthmatics

This study used ASM-Asthma (ASM-A (Ark)) (n =  11) and ASM-Non-Asthma (ASM-NA (Ark)) (n =  7) derived from postmortem human lungs obtained by the Arkansas Children’s Research Institute Lung Cell Biology Laboratory as previously described [16]. The diagnosis of asthma was determined by a physician diagnosis of asthma and the use of asthma medications in the medical record. In contrast, the non-asthma group did not have a physician diagnosis of asthma, and did not use asthma medications. The University of Arkansas for Medical Sciences and the University of California San Diego Institutional Review Boards determined that the study of post-mortem human lungs was not human subject’s research.

### ASM isolation from postmortem lungs of asthmatics and non-asthmatics

To isolate a pure population of ASM from postmortem human lungs, we used methods previously described in our lab [5,17] that result in >  90% pure population of ASM. In brief, bronchial airways of postmortem lung were dissected from surrounding tissue, cut into ~  1 cm pieces, and transferred to 6-well cell culture plates for cell attachment and growth in smooth muscle cell complete media (ScienCell), which included smooth muscle cell-specific medium, smooth muscle cell growth factors, 10,000 U/mL penicillin, and fetal bovine serum. After detection of cell outgrowth onto the plate ( ~ 7 days), bronchial tissue was removed. ASM cells were identified by morphology and RT-qPCR for α-smooth muscle actin after 3-4 weeks of culture as previously described [5]. ASM cells were passaged weekly and used in experiments within the 3^rd^ -7^th^ passage. For experiments, ASM cells were initially transferred to a standard 6-well plate (1x10^6^ ASM per well in 2 mL media) and incubated at 37°C for 24 hrs prior to the start of an experiment.

### Levels of PGAP3 mRNA expression by RT-qPCR in ASM from asthma vs non-asthma

RNA was extracted from (ASM-A (Ark)) and (ASM-NA (Ark)) with a RNeasy Mini Kit (50, Cat No. 74104, Qiagen) as previously described in this laboratory [5]. DNAse digestion (DNA digestion kit: Qiagen, Cat #79254) was included to control for DNA contamination. In brief, RNA quantity and quality were measured using a NanoDrop 2000/2000c spectrophotometer (ND2000, Thermo Fisher Scientific). RNA samples (1 µgram RNA/sample) were added to cDNA EcoDry Premix (Cat No. 639543, TaKaRa) and reverse transcribed using an Eppendorf Mastercycler (MilliporeSigma) set to 42°C for 60 min and 70°C for 10 min. 1 μL cDNA was used for real-time quantitative polymerase chain reaction (RT-qPCR) performed as previously described in this laboratory [5] to quantitate levels of PGAP3 mRNA (PGAP3 primer Assay ID: Hs.PT.58.19820740, IDT) expression in ASM-A (Ark) and ASM-NA (Ark). The delta delta Ct cycle threshold method was used to determine the expression of PGAP3 after normalization to GAPDH (IDT, Cat #Hs.PT.39a.22214836) and the results analyzed with an unpaired student t-test as previously described in this laboratory [5].

### Source of ASM cells for in vitro transfection of ASM with PGAP3 plasmid

For *in vitro* studies of ASM (RNA-seq, bioinformatics, ASM proliferation, ASM contractility), a pure population of normal ASM (ASM-NA (SC)) were purchased from ScienCell to have sufficient number of cells for study. In addition, we wanted to transfect ASM-NA (SC) with PGAP3 plasmid as non-asthmatic ASM express very low levels of PGAP3 mRNA allowing us to investigate how increased PGAP3 regulates ASM genes, pathways, and function.

### Transfection of ASM cells with PGAP3 plasmid or empty vector

ASM-NA (SC) (10^6^ cells per sample) were transfected with either PGAP3 plasmid (or as a control the empty plasmid not containing PGAP3) to simulate increased PGAP3 expression observed in ASM-A (Ark) to study the downstream effects of increased PGAP3 on gene expression and ASM function. We used the pCMV6 plasmid vector (OriGene, Cat #RC202289), which has a CMV promoter which induces constitutive expression of full length PGAP3 already cloned into the vector. As a control we used the empty pCMV6 plasmid vector (OriGene, Cat #PS1000001) which does not contain PGAP3. ASM cells were transfected using methods previously described in this laboratory to study the effects of transfected ORMDL3 and GSDMB on gene expression and cell function [17,18]. In brief, ASM-NA (SC) cells were transfected in 6-well plates (Fisher Scientific, Cat # 152035) with a 250 μL solution containing the following final volumes per well: 1 μg (10 μL) pCMV6 plasmid vector with or without PGAP3, 15 μL lipofectamine (Invitrogen), and 225 μL DMEM media (Opti-MEM, Cat No. 11058021, Invitrogen). For transfection, an additional 750 μL DMEM media was added to each well to prevent evaporation for a final volume of 1 mL per well. After 24 or 48 hrs of transfection, ASM-NA (SC) cells were processed for RNA-sequencing.

### Bulk RNA-sequencing of PGAP3 plasmid or empty vector transfected ASM to identify PGAP3 regulated gene expression

PGAP3 plasmid, or expression vector control, transfected ASM-NA(SC) samples (n =  3 per group) were sent to GENEWIZ (Azenta Life Sciences) for RNA-sequencing. Using their automated workflow, sequence reads were processed using quality assessment, trimming (Trimmomatic v.0.36), genome mapping (human reference genome GRCh38, STAR aligner v.2.5.2b), and calculation of unique gene hit counts filtered for reads that fell within exon regions (Subread package v.1.5.2). Differential expression analysis was then performed (DESeq2) with significance (*p* <  0.05) and log2 fold change (FC) determined via the Wald test.

### Comparison of PGAP3-induced genes in ASM to other data sets

We compared the results of PGAP3-induced genes in ASM-NA (SC) to several other data sets including PGAP3 induced genes in NHBE [14], genes expressed in ASM data sets [19–21], and to genes expressed by asthma patients in the UK BioBank [22]. We did not use any mathematical formula or software to compare results of our study of ASM to that of different data sets [14,19–22]. We listed the upregulated genes expressed in each of the other data sets separately in an Excel file. Each of these upregulated genes in the different data sets were then inspected to determine whether they were present in our data set. In this manner we were able to build a list of genes that were common to our dataset and any of the comparison data sets.

### Comparison of PGAP3-induced genes in ASM to PGAP3-induced genes in NHBE

To determine whether PGAP3 induced the same, or a different, profile of genes in ASM compared to in NHBE [14], we compared the profile of genes induced by PGAP3 in ASM-NA (SC) cells in this study, to genes induced by PGAP3 in NHBE in a prior study from this laboratory [14].

### Comparison of PGAP3-induced genes in ASM to genes in asthma ASM data sets

PGAP3-transfected gene expression from ASM-NA (SC) cells was compared to genes upregulated in ASM from asthmatics in three different asthmatic ASM data sets. The three different asthmatic ASM data sets include the data set from (1) Alexandrova et al., (2016) [19] which includes differentially expressed genes, determined by cap-analysis of gene expression, of asthmatic smooth muscle compared to non-asthmatic controls (n =  99 upregulated genes), (2) Banerjee et al., (2021) [20] which contain RNA-sequencing results of differentially expressed genes in asthmatic vs. non-asthmatic ASM (n =  83 upregulated genes), and (3) Yick et al., (2014) [21] which also contains RNA-sequencing results of differentially expressed genes in asthmatic vs. non-asthmatic ASM from non-atopic patients (n =  106 upregulated genes).

### Comparison of PGAP3-induced genes in ASM to asthma reference data set

The asthma reference data set was included to determine the relationship between PGAP3-induced genes in ASM-NA (SC) cells with those known to be associated with asthma, as well as to find potentially new relationships with those induced by PGAP3. The asthma reference data set we utilized is composed of genes associated with 61 asthma-specific loci identified in a genome-wide association study of samples from 37,846 self-reported asthma patients in the UK BioBank [22]. Protein-coding genes associated with these loci were searched for in the National Center for Biotechnology Information (NCBI) Gene Database (https://www.ncbi.nlm.nih.gov/gene/?term=). For the search, the advanced search function ‘Default Map Location’ was used and the results were filtered for current and protein-coding genes (search completed on March 28, 2023). The results included 2,326 protein-coding genes that were used as the asthma reference data set.

### GPI-anchored proteins among PGAP3-induced genes in ASM

To determine if PGAP3 transfection influences the expression of GPI-APs, GPI-APs were searched for among differentially expressed genes (*p* <  0.05). UniProt (https://www.uniprot.org) [23] was used to compile a reference list of GPI-APs. This list was obtained by searching for “GPI-anchored protein” and filtering for Human, resulting in a reference list of 168 GPI-APs (compiled on August 23, 2023). The search list identified GPI-AP, but also identified several proteins that modify or bind to GPI-AP and are not themselves GPI-AP (PGAP3, EPHA5, ACE, EPHA7). Thus, these proteins that modify or bind to GPI-AP are not included in our list of GPI-AP regulated by PGAP3.

### Bioinformatic analysis of PGAP3-induced genes

Beyond differential expression analysis of PGAP3-transfected vs. expression vector control ASM-NA (SC), we performed further bioinformatic analyses to determine mechanisms of interest related to increased PGAP3 expression and asthma. Specifically, we performed overrepresentation, gene set enrichment, and network topology analysis with the web-based application WebGestalt (https://www.webgestalt.org) [24]. Overrepresentation was completed using the asthma reference data set [22] as background to determine any connections between PGAP3 to asthma-associated genes, as determined by genome-wide association. Additionally, for both overrepresentation and gene set enrichment analyses, results were focused on significant Gene Ontology (GO) biological processes of non-redundant genes and Kyoto Encyclopedia of Genes and Genomes (KEGG) pathways. The protein-protein interaction network from BIOGRID was used for network topology-analysis to identify highlighted seeds.

### ASM proliferation in PGAP3-transfected cells

An ASM proliferation assay using a BrdU cell proliferation ELISA (Exalpha Biologicals, Inc.) was performed in PGAP3 plasmid, or expression vector control, transfected ASM-NA (SC) cells (n =  8 per group) as previously described in this laboratory [5]. In brief, ASM-NA (SC) cells transfected with either PGAP3 plasmid, or expression vector without PGAP3 (2 x 10^4^ cells per well in a 96-well plate) were incubated for 24 hrs, and then incubated for an additional 18 hrs with BrdU (with or without 10% fetal bovine serum as a stimulus for ASM proliferation). BrdU absorbance was measured by ELISA with results expressed both as a) absolute values of BrdU absorbance, as well as b) a BrdU percentage change in PGAP3-transfected samples compared to vector control.

### ASM contractility in PGAP3-transfected cells

An ASM contractility assay was performed in PGAP3 plasmid, or expression vector control, transfected ASM-NA (SC) cells (n =  3 per group) as previously described in this laboratory [5]. In brief, a smooth muscle gel contraction assay (Cell Biolabs, Inc.) was used as an indicator of contractility to histamine. ASM were seeded in LPS-free collagen gels (2 ×  10^5^ cells/well) (Advanced BioMatrix) and incubated for three days in smooth muscle-specific media with 1.8 mM Ca^2 +^ (ScienCell). ASM-NA (SC) cells were then cultured with a contractile agonist (100 μM histamine) or media control with measurements of contractility taken at the following time points: 0, 15, 30, 45, and 60 minutes. ASM cell contraction was quantified by comparing the area of the ASM prior to histamine to the area of the ASM after histamine incubation (i.e., percentage of contracted gel area/ total area in mm) using a Bio-Rad ImageDR transilluminator and Versadoc scanner (Bio-Rad Laboratories) and analyzed with the accompanying software [5].

### Statistical analysis

Unpaired t-tests were used to analyze the results between levels of PGAP3 mRNA expression by RT-qPCR in ASM from ASM-A (Ark) vs ASM-NA (Ark) as well as ASM proliferation and contractility in PGAP3-Transfected ASM-NA (SC) cells. For all bioinformatic analyses, Benjamin-Hochberg multiple test adjustment and a false discovery rate (FDR) level of <  0.05 were used, as applicable.

## Results

### Increased PGAP3 expression in human asthmatic airway smooth muscle

**Fig 1:**
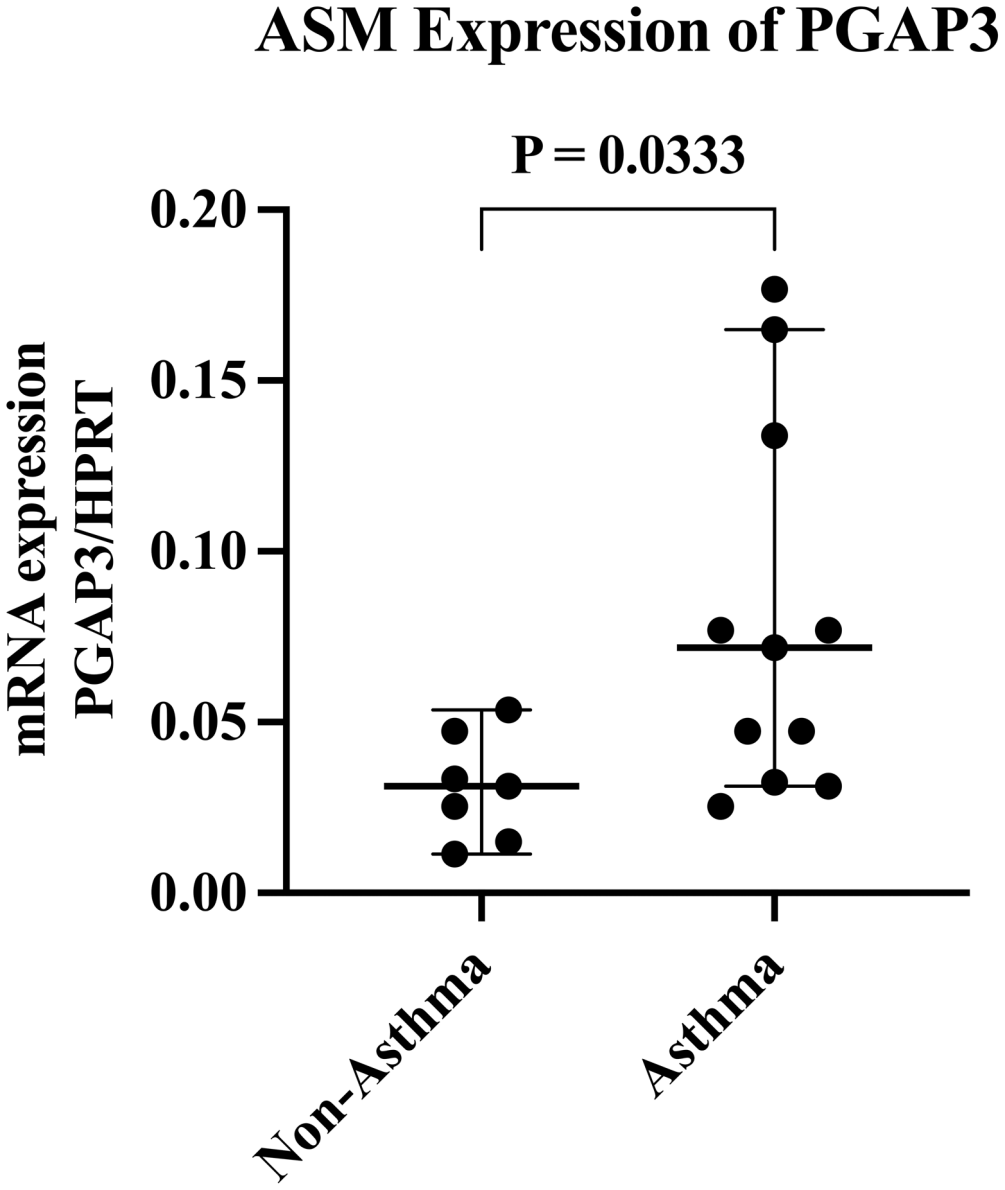
PGAP3 expression in human ASM-A (Ark) and ASM-NA (Ark). Levels of PGAP3 mRNA quantitated by RT-qPCR in ASM-A (Ark) (n =  11) were significantly higher than in ASM-NA (Ark) (n =  7) (*p* =  0.033).

ASM-A (Ark) derived from postmortem human lung bronchi showed significantly higher levels of PGAP3 mRNA expression compared to ASM-NA (Ark) as quantitated by RT-qPCR (*p* =  0.033) (Fig 1).

### RNA-seq: PGAP3 upregulated genes in ASM

RNA-sequencing was used to determine if increased PGAP3 expression induced by plasmid PGAP3 transfection of ASM-NA (SC) cells influences the expression of genes that contribute to asthma. In total, 5 genes at 24 hrs, and 1,445 genes at 48 hrs were differentially upregulated in PGAP3-transfected ASM-NA (SC) cells compared to expression vector controls (*p* <  0.05) (Table 1). Full lists of differentially upregulated genes at 24 hrs are provided in S1 Table in S1 File and at 48 hrs is provided in S2 Table in S1 File. Because of the larger number of genes upregulated by PGAP3 at 48 hrs, subsequent analysis focused on the 48-hour timepoint. Four of the 1,445 upregulated genes at 48 hrs were uncharacterized (denoted in S2 Table in S1 File with “NA”) and were removed from further analysis.

**Table 1:** PGAP3-Regulated Genes in Normal Human Bronchial ASM.

	24 Hours	48 Hours
**Upregulated**	5	1,445
**Downregulated**	23	1,326

PGAP3 vs. expression vector control expressed genes in ASM-NA (SC) that achieved significance at an alpha level of 0.05 were included. The data set includes 10 of the uncharacterized genes expressed at 48 hours (four upregulated, six downregulated) that were given a gene symbol of “NA.” These genes are included in the totals provided here but were removed from subsequent analyses. Only genes expressed 48 hrs after PGAP3 transfection were used in subsequent analyses.

### RNA-seq: PGAP3 downregulated genes in ASM

In total, 23 genes at 24 hrs, and 1,326 genes at 48 hrs were differentially downregulated in PGAP3-transfected ASM-NA (SC) compared to empty vector controls (*p* <  0.05) (Table 1). Full lists of differentially downregulated genes at 24 hrs are provided in S1 Table in S1 File and at 48 hrs is provided in S2 Table in S1 File. Six of the 1,326 downregulated genes at 48 hrs were uncharacterized (denoted in S2 Table in S1 File with “NA”) and were removed from further analysis.

### Top 5 upregulated genes induced by PGAP3 in ASM

The top 5 upregulated genes after PGAP3 transfection in ASM-NA (SC) include genes previously associated to asthma, including GATA3 (log_2_ fold change (FC) =  4.99) [25] and ALOX5 (FC =  4.88) [26] at 48 hrs (Table 2), as well as Alcohol Dehydrogenase 1B (Class I) Beta Polypeptide (ADH1B) (log_2_ fold change (FC) =  1.04) [27] at 24 hrs (Table 2).

**Table 2:** Top 5 PGAP3-Upregulated Genes in Normal Human Bronchial ASM.

24 Hours			48 Hours		
Rank	Gene	log2 Fold Change (Adj.p-value^*^)	Rank	Gene	log2 Fold Change (Adj.p-value^*^)
1	PGAP3 (Post-GPI Attachment to Proteins Phospholipase 3)	7.43 ( < 0.001)	1	NCMAP (Non-Compact Meylin Associated Protein)	6.88 ( < 0.001)
2	GH1 (Growth Hormone 1)	1.60 ( < 0.001)	2	FOXI1 (Forkhead Box I1)	6.07 ( < 0.001)
**3**	**GPBAR1** **(G Protein-Coupled Bile Acid Receptor 1)**	**1.05** **( < 0.001)**	3	AL132857.2 (Uncharacterized Gene)	5.22 ( < 0.001)
4	ALPK2 (Alpha Kinase 2)	1.04 ( < 0.001)	4	**GATA3** **(GATA Binding Protein 3)**	**4.99** **( < 0.001)**
**5**	**ADH1B** **(Alcohol Dehydrogenase 1B (Class I), Beta Polypeptide)**	**1.04** **( < 0.001)**	5	**ALOX5** **(Arachidonate 5-Lipoxygenase)**	**4.88** **( < 0.001)**

* =  Benjamin-Hochberg adjusted *p*-values. Bold text =  select PGAP3-upregulated genes associated with asthma that are discussed in text.

### Top 5 downregulated genes induced by PGAP3 in ASM

The top 5 downregulated genes after PGAP3 transfection in ASM-NA (SC) include genes such as IL21R (FC =  -1.81) at 24 hrs, and MMP12 (FC =  -7.79) at 48 hrs (Table 3). The IL21R is involved in TH2 differentiation [28] and MMP12 has been proposed as a diagnostic marker for eosinophilic asthma [29].

**Table 3:** Top 5 PGAP3-Downregulated Genes in Normal Human Bronchial ASM.

24 Hours			48 Hours		
Rank	Gene	log2 Fold Change (Adj.p-value^*^)	Rank	Gene	log2 Fold Change (Adj.p-value^*^)
1	**IL21R** **(Interleukin 21 Receptor)**	**-1.81** **( < 0.001)**	1	GJA4 (Gap Junction Protein Alpha 4)	-7.84 ( < 0.001)
2	IBSP (Integrin Binding Sialoprotein)	-1.71 ( < 0.001)	2	**MMP12** **(Matrix Metallopeptidase 12)**	**-7.79** **( < 0.001)**
3	MT1G (Metallothionein 1G)	-1.64 ( < 0.001)	3	DHRS9 (Dehydrogenase/Reductase 9)	-7.69 ( < 0.001)
4	CSF3 (Colony Stimulating Factor 3)	-1.62 ( < 0.001)	4	PPP1R1B (Protein Phosphatase 1 Regulatory Inhibitor Subunit 1B)	-7.68 ( < 0.001)
5	MIR3142HG (MIR3142 Host Gene)	-1.60 ( < 0.001)	5	SPANXN4 (SPANX Family Member N4)	-7.24 ( < 0.001)

* =  Benjamin-Hochberg adjusted p-values. Bold text =  select PGAP3-upregulated genes associated with asthma that are discussed in text.

### PGAP3 upregulated genes also found in PGAP3-transfected NHBE

We have previously reported that transfection of NHBE with PGAP3 upregulates genes and pathways of potential importance to asthma [14]. We were interested to determine whether PGAP3 regulated the same pathways in both ASM and NHBE and/or whether there were unique pathways regulated by PGAP3 in ASM and NHBE. There were 59 genes in common that were upregulated by PGAP3 in both PGAP3-transfected ASM-NA (SC) and PGAP3-transfected NHBE. The top 10 PGAP3 upregulated genes in both ASM and NHBE are provided in Table 4 and the full list is provided in S3 Table in S1 File. Two of the genes upregulated by PGAP3 in both ASM and NHBE include BMP4 (FC =  3.12) and MMP13 (FC =  3.03). Levels of BMP4 have correlated with FEV1 and FEV1/FVC in asthma [30], while in vitro studies demonstrate that MMP13 limits bronchial epithelial repair as it is a protease that degrades fibrinogen and matrix proteins essential for epithelial repair [31]. Limiting bronchial epithelial repair following respiratory virus infection may contribute to the persistence of asthma.

**Table 4:** The Top 10 PGAP3-Upregulated Genes in Normal Human Bronchial ASM Found in PGAP3-Regulated NHBE Reference Data Set.

Gene	PGAP3-Induced Fold Change (log2)
PGAP3	4.86
RP1L1	3.95
AC008556.1	3.55
ST8SIA4	3.33
**BMP4**	**3.12**
**MMP13**	**3.03**
SP6	2.94
AL365181.3	2.87
KRT75	2.86
PART1	2.81

PGAP3-upregulated genes that achieved significance at an alpha level of 0.05 were included. Bold text =  select PGAP3-upregulated genes associated with asthma that are discussed in text.

### PGAP3 upregulated genes also found in ASM asthma reference data sets

We examined whether any of the PGAP3 upregulated genes we detected in ASM-NA (SC) could also be detected at increased levels in ASM from asthmatics using RNA-seq data sets from three prior studies [19–21] of genes upregulated in asthma compared non-asthma control ASM. We identified 15 genes that were upregulated by PGAP3 in ASM-NA (SC) that were also detected in one of the three asthma ASM reference data sets (Table 5). This suggests that PGAP3 has the ability to induce multiple genes in ASM that can be detected in ASM from asthmatics as opposed to non-asthmatics. In comparing PGAP3-induced genes in ASM in this study to genes expressed by ASM in asthmatics in the study of Yick et al. (2014), we identified two genes (SERPINB5: FC =  3.50 and SCUBE3: FC =  1.84) regulated by PGAP3 in ASM that were detected at higher levels in ASM from asthmatics compared to non-asthmatic ASM from healthy, non-atopic subjects (Table 5). SERPINB5, which is predicted to be involved in extracellular matrix organization (https://www.alliancegenome.org/gene/HGNC:8949#summary), had the highest fold change among these genes. At present it is unclear why the three different ASM RNA-seq datasets (asthma vs control ASM) have different genes upregulated in their asthma ASM data set, but it could be due to differences in the three studies in the severity of asthmatics studied, medications used to treat asthma, methods used to isolate ASM, differences in human reference genome, as well as other at present unknown factors.

**Table 5:** Compiled PGAP3-Upregulated Genes in Normal Human Bronchial ASM Found in ASM Asthma Reference Data Sets.

Gene	PGAP3-Induced Fold Change (log2)
**SERPINB5** ^**(2)**^	**3.50**
SLITRK6^(1)^	3.48
TLR5^(1)^	3.42
EYA1^(1)^	2.91
KIAA1671^(1)^	2.56
CCDC88C^(1)^	2.35
ZNF608^(1)^	1.94
PCDH19^(1)^	1.87
SCUBE3^(2)^	1.84
B4GALT6^(1)^	1.77
SLC4A3^(1)^	1.56
ZC3H6^(1)^	1.50
ACACB^(1)^	1.19
INSR^(1)^	1.05
LINC00886^(1)^	1.00

PGAP3-upregulated genes that achieved significance at an alpha level of 0.05 were included. Bold text =  select PGAP3-upregulated genes associated with asthma that are discussed in text. ^(1)^ =  genes found in the smooth muscle asthma reference data set from Banjeree et al. (2021). ^(2)^ =  genes found in the smooth muscle asthma reference data set from Yick et al. (2014).

### PGAP3 upregulated genes in ASM found in asthma reference data set

We identified that PGAP3 upregulated 151 ASM-NA (SC) genes that were also detected in a GWAS asthma reference data set [22]. The top 10 PGAP 3 upregulated genes in ASM also detected in a GWAS asthma reference data set are shown in Table 6 and the full list of 151 genes is provided in S4 Table in S1 File). Of these 151 PGAP3 upregulated genes found in both our ASM-NA (SC) data set and in the Asthma Reference data set, GATA3 had the highest overall fold change (FC =  4.99) (Table 6 in S1 File). GATA3 is a transcription factor which regulates T2 responses in asthma [32].

**Table 6:** PGAP3-Upregulated Genes in Normal Human Bronchial ASM Found in the UK BioBank Asthma Reference Data Set.

Gene	PGAP3-Induced Fold Change (log2)
**GATA3**	**4.99**
PGAP3	4.86
KRT15	4.33
KCNN3	4.17
INAVA	3.68
**SERPINB5**	**3.50**
PLXDC1	3.17
ENTPD2	2.95
CHMP4C	2.95
SP6	2.94

PGAP3-upregulated genes that achieved significance at an alpha level of 0.05 were included. Bold text =  select PGAP3-upregulated genes associated with asthma that are discussed in text.

### PGAP3 upregulated genes in ASM, NHBE, and an asthma reference data set

An overall comparison between the PGAP3 upregulated genes in ASM-NA (SC), PGAP3 upregulated genes in NHBE, and the GWAS asthma reference data set was performed to identify genes in common in all three datasets of relevance to asthma. This comparison found seven genes in common among the three data sets (Table 7), which includes PGAP3 (FC =  4.86) and the transcription factor SP6 (FC =  2.94). SP6 belongs to a family of transcription factors that contain zinc finger DNA-binding domains and bind to GC-rich sequences [33].

**Table 7:** PGAP3-Upregulated Genes Found in PGAP3-Transfected ASM-NA (SC), in PGAP3-Transfected NHBE, and in the UK BioBank Asthma Reference Data Sets.

Gene	PGAP3-Induced Fold Change (log2)
PGAP3	4.86
**SP6**	**2.94**
IL24	1.36
RMI2	1.13
CFB	1.11
HLA-B	1.06
CCL5 (RANTES)	1.04

PGAP3-upregulated genes that achieved significance at an alpha level of 0.05 were included. Bold text =  select PGAP3-upregulated genes associated with asthma that are discussed in text.

### PGAP3 regulation of GPI-anchored proteins

As PGAP3 is a GPI anchor-remodeling gene, we examined whether PGAP3 upregulated levels of GPI-anchored proteins. Of the 168 total GPI-APs and/or proteins that modify or bind to GPI-AP we identified on our UniProt search, there were five GPI-AP-encoding genes induced by PGAP3 in ASM-NA (SC) (Table 8). These include genes that may influence cell adhesion (Mesothelin (MSLN): FC =  2.44) as well as two genes influencing vascular function, including inhibiting angiogenesis LY6/PLAUR Domain Containing 1 (LYPD1), FC =  2.17), and promoting vascular development Ephrin A5 (EFNA5): FC =  1.88).

**Table 8:** PGAP3 GPI-Anchored Proteins mRNA induced in Normal Human Bronchial ASM.

24 Hours			48 Hours		
Gene	Log2 Fold Change	Function	Gene	Log2 Fold Change	Function
NA			MSLN	2.44	Cell Adhesion
			LYPD1	2.17	Inhibits Angiogenesis
			FOLR1	2.08	Folate Binding
			PRSS21	1.99	Tumor Progression
			EFNA5	1.88	Vascular Development

PGAP3-upregulated genes that achieved significance at an alpha level of 0.05 were included. NA =  no GPI-anchored proteins found after 24 hours of PGAP3 transfection.

### Bioinformatic analyses of PGAP3-induced ASM genes

All highly significant PGAP3-induced genes in ASM (*p* <  0.05) were included in gene set enrichment, overrepresentation, and network topology analyses to determine the presence of any PGAP3 regulated pathways.

Gene set enrichment analysis showed that all (10 total) significant KEGG pathways were downregulated, which include proinflammatory signaling pathways related to TNFα and IL-17 signaling (S1A Fig). The only two significant Gene Ontology biological processes were related to skin and epidermis development, while there were also downregulated processes related to mitotic cell cycle phase transition and chromosome segregation (S1B Fig). These findings were unexpected considering the positive relationship of PGAP3 expression and Asthmatic ASM in ASM-A (Ark) (Fig 1).

We also performed an overrepresentation analysis whereby the asthma reference data set was used as background. However, this analysis produced limited results, as there was only one significant KEGG pathway (cytokine-cytokine receptor interaction, S2A Fig) and five significant Gene Ontology biological processes (muscle cell proliferation, response to chemokine, circulatory system process, angiogenesis, and positive regulation of cell motility, S2B Fig). Our network topology analysis also produced limited results since there were only two highlighted seeds with FC ≥  2.0 (MDFI and RHOU, S5 Table in S1 File).

### Effects of increased PGAP3 in ASM on ASM proliferation and contractility

**Fig 2:**
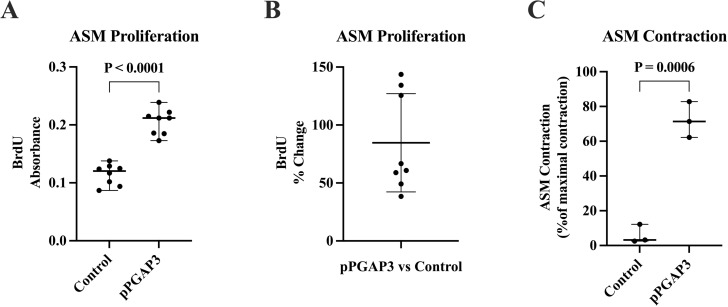
PGAP3 induced ASM Proliferation and contractility. 2A: ASM-NA (SC) transfected with PGAP3 plasmid had significantly increased proliferation compared to ASM-NA (SC) transfected with an expression vector control that did not contain PGAP3 as quantitated in a BrdU proliferation assay (p < 0.0001) (n = 8/group). 2B: Results of the same BrdU ASM proliferation assay experiments in Fig 2A (where results were expressed as absolute BrdU absorbance values) were also expressed as BrdU percentage change in Fig 2B (from baseline value of vector control BrdU to PGAP3 plasmid BrdU =  84.7 ±  15.0% change, Mean ± SEM). 2C: Levels of ASM contraction (using a smooth muscle gel contraction assay) [5] were significantly higher in ASM-NA (SC) transfected with PGAP3 plasmid compared to ASM-NA (SC) transfected with an expression vector control that did not contain PGAP3 (p =  0.0006).

PGAP3-transfected ASM-NA (SC) had significantly increased proliferation compared to expression vector controls (*p* <  0.0001) (Fig 2A, 2B). PGAP3-transfected ASM-NA (SC) also had significantly greater contractility compared to expression vector controls (*p* =  0.0006) (Fig 2B).

## Discussion

The importance of PGAP3, a gene localized to chromosome 17q12-21, to the pathogenesis of asthma is suggested from genetic association studies demonstrating a link between PGAP3 SNPs and increased PGAP3 expression [1–3] as well as to asthma exacerbations [10], asthma severity [7], and asthma susceptibility [6]. As it is not known whether in asthma PGAP3 is expressed in ASM (a key cell in the airway in asthma regulating bronchoconstriction, airway hyperreactivity, and airway remodeling), in this study we made the novel observation that PGAP3 is significantly expressed in ASM in asthma, and that ASM derived from the lungs of asthmatics express significantly higher levels of PGAP3 compared to non-asthmatics. In addition, this study demonstrated that when ASM from non-asthmatics were transfected with PGAP3, the increased levels of PGAP3 in ASM increased ASM proliferation and ASM contractility, which are cardinal features of ASM in asthma [15]. Moreover, RNA-seq studies demonstrated that increased PGAP3 expression in ASM increased levels of genes previously linked to asthma including GATA3 [25] and ALOX5 [26]. Overall, these studies suggest that increased PGAP3 expression in ASM plays an important functional role in ASM in contributing to airway hyperreactivity (AHR) and the pathogenesis of asthma.

The ability of chromosome 17q12-21 gene PGAP3 to regulate ASM function has previously been noted with other chromosome 17q12-21 genes including ORMDL3 [34] and GSDMB [17]. For example, in vitro studies of ASM cells isolated from the bronchi of transgenic mice which express increased levels of human ORMDL3 selectively in smooth muscle cells (i.e. hORMDL3^Myh11eGFP-cre^ mice) demonstrated that these ASM had increased contractility to histamine, ASM hypertrophy (quantitated by FACS and image analysis), ASM hyperplasia (assessed by BrdU incorporation), and expressed increased levels of tropomysin proteins TPM1 and TPM4 [5]. In vivo studies of hORMDL3^Myh11eGFP-cre^ mice demonstrated that they had a spontaneous increase in ASM and AHR. ORMDL3 expression in ASM thus induces changes in ASM (hypertrophy, hyperplasia, increased contractility), which may explain the contribution of ORMDL3 to the development of AHR in childhood onset asthma. We have also previously demonstrated the potential of GSDMB another chromosome 17q12-21 localized gene to contribute to airway remodeling and AHR [17]. For example, increased expression of GSDMB in primary human bronchial epithelium increased expression of genes important to both airway remodeling [TGFβ1, 5-lipoxygenase (5-LO)] and AHR (5-LO). Interestingly, hGSDMB^Zp3-Cre^ mice expressing increased levels of the human GSDMB transgene showed a significant spontaneous increase in AHR and a significant spontaneous increase in airway remodeling, with increased smooth muscle mass and increased fibrosis in the absence of airway inflammation [17]. In this study we have identified a third gene on chromosome 17q12-21 (i.e., PGAP3) that can increase ASM contractility and proliferation. The mechanisms by which each of these three genes (OMDL3, GSDMB, and PGAP3) contribute to increased ASM contractility and AHR is likely to differ as studies of ORMDL3 suggest an important role for ASM sarcoplasmic reticulum Ca^2 +^ ATPase 2b (SERCA2b) [18], while studies of GSDMB suggest an important role for epithelial TGFβ1, and 5-lipoxygenase (5-LO) in GSDMB-mediated ASM contractility and remodeling [17]. At present the pathways that mediate PGAP3-induced ASM proliferation and contractility are not known.

Although the pathways that mediate PGAP3 induced ASM proliferation and contractility are not known, RNA-Sequencing of ASM expressing PGAP3 identified four genes significantly upregulated by PGAP3 that have known relevance with asthma (i.e., GATA3, ALOX5, BMP4, and SERPINB5). The most prominent asthma-related PGAP3-upregulated gene in ASM found in our data set is GATA3, which was the fourth highest expressed gene (FC =  4.99) induced by PGAP3 in ASM. GATA3 has more well-known expression in T cells and epithelial cells (https://www.proteinatlas.org/ENSG00000107485-GATA3) than ASM. GATA3 has previously been shown to have strong links to allergic asthma and the promotion of Th2 differentiation and the resulting release of cytokines regulating ASM contractility, including IL-13 [32] and IL-5 [25]. At present, the function in ASM of GATA3 (induced by PGAP3) is unknown as ASM is not a known source of IL13 and IL5.

The second most prominent asthma-related PGAP3-upregulated gene in ASM found in our data set is ALOX5 (FC =  4.88). ALOX5 has previously been shown to contribute to asthma in ASM through leukotrienes derived from arachidonic acid metabolism [26]. In addition to PGAP3 upregulating ALOX5 in ASM, GSDMB, another chromosome 17q12-21 gene, upregulates ALOX5 in NHBE, and knocking down ALOX5 reduced GSDMB-induced TGFβ1 expression [17]. Thus, induction of ALOX5 in NHBE has functional effects on GSDMB-induced TGFβ1 expression. At present it is not known whether PGAP3 induction of ALOX5 has functional effects in ASM and requires further study. Interestingly, in the current PGAP3-transfected ASM data set, ALOX5 was upregulated (FC =  4.88), and TGFβ1 was downregulated (FC =  -1.45), suggesting that in ASM there may be a relationship between ALOX 5 and TGFβ1 that affects the intracellular levels of TGFβ1.

Two other notable genes upregulated by PGAP3 in ASM are BMP4 (FC =  3.12) and SERPINB5 (also known as Maspin, FC =  3.50), which each have studies supporting their potential role in asthma. For example, BMP4 has significant negative correlations to FEV1 and FEV1% [30]. This indicates that with higher expression of BMP4 there is a correlation to reduced lung function that is a consistent finding in asthmatics that experience asthma exacerbations [35]. Reduced lung function induced by BMP4 may relate to BMP functioning as a ligand of TGFβ that decreases extracellular matrix production in lung fibroblasts [36]. SERPINB5 is the highest expressed PGAP3-induced ASM gene found among the ASM data sets (found in Yick et al. (2014)) [21]. Levels of SERPINB5 are increased in bronchoalveolar lavage fluid in a mouse model of asthma following a house dust mite challenge [37]. The biologic importance of SERPINB5 to the pathogenesis of asthma is at present unknown, but a study [37] has shown that mechanical compression (as might occur with bronchoconstriction) increases SERPINB5 expression in bronchial epithelial cells *in vitro*.

Our Bioinformatic analysis of the 1,445 PGAP3 upregulated genes in the ASM dataset identified that approximately 10.5% of these genes (i.e., 151 genes) were found in a GWAS asthma reference data set [22]. For comparison, PGAP3 overexpression in NHBE identified 62 genes in a GWAS asthma reference data set [14]. Thus, the overall number of genes induced by PGAP3 in ASM and identified in a GWAS asthma reference dataset (151 genes) is greater than the 62 genes induced by PGAP3 in NHBE and identified in the same GWAS asthma reference dataset. This suggests that PGAP3 overall upregulated many more genes in ASM than in airway epithelium of importance to asthma.

Our Bioinformatic analysis of the upregulated genes induced by PGAP3 in ASM identified several downregulated pathways related to inflammation (e.g., TNF signaling, IL-17 signaling) and cell signaling (e.g., mitotic cell cycle phase transition, chromosome segregation). Additionally, while muscle cell proliferation and angiogenesis were significant biological processes in the overrepresentation analysis, most of the genes involved in these processes were downregulated (4 upregulated genes and 6 downregulated genes involved in muscle cell proliferation, 6 upregulated genes and 12 downregulated genes involved in angiogenesis). Therefore, none of the pathways identified by Bioinformatic analysis of the RNA-seq data sets were able to explain the pathways induced by PGAP3 in ASM that induce ASM proliferation and/or ASM contractility, which will require further study.

We have examined the effect of increased expression of PGAP3 in two cell types important to the pathogenesis of asthma, i.e., ASM cells in this study as well as in bronchial epithelial cells in a prior study [14]. The range of levels of PGAP3 mRNA expression in ASM and epithelium from asthmatics is similar in ASM and epithelium. Taken together the results of these studies suggest that PGAP3 expression in either ASM cells or epithelial cells are likely to both be important in regulating different features of asthma based both on the difference in anatomical location of these cells in the airway, as well as based on the different function of these cells. For example, epithelial cells line the interior surface of the airway which is the site of contact with inhaled respiratory viruses relevant to asthma. Thus, PGAP3 expression in epithelial cells could play a role in innate immune response to viruses (such as rhinovirus) which trigger asthma exacerbations. Our previous studies of epithelial cells have demonstrated that PGAP3 regulates genes important in the innate immune response to rhinovirus [14]. In contrast, ASM cells surrounding the airway would not be the initial cells exposed to viruses in the airway, and PGAP3 expression in ASM would rather be important in influencing ASM functions such as contraction which we have noted in this study, resulting in bronchoconstriction that is an airway response noted in asthma exacerbations. The mechanism by which PGAP3 regulates increased ASM contractility is at present unknown as neither our Bioinformatic analysis of contractility pathways, nor our pilot RT-qPCR studies for candidate ASM contractility-associated proteins/signaling genes (i.e., SERCA2a, SERCA2b) have found any differences between PGAP3 transfected ASM and control vector transfected ASM.

PGAP3 appears to regulate different pathways in ASM and epithelial cells. For example, our RNAseq studies demonstrate that increased expression of PGAP3 in epithelial cells upregulates 654 genes, while increased expression of PGAP3 in ASM upregulates 1,445 genes. When we compare the genes upregulated by PGAP3 in epithelium to the genes upregulated by PGAP3 in ASM, only a minority of the same PGAP3 regulated genes are upregulated in common by PGAP3 in both cell types (59 genes regulated in common). Our bionformatic analysis of epithelial cells identified that PGAP3 upregulated several pathways including antiviral and innate immune responses. In contrast, PGAP3 did not upregulate these pathways in ASM. Thus, at present our data suggests that PGAP3 upregulates different pathways in epithelial cells and ASM. However, this data does not exclude that PGAP3 upregulates a similar pathway in epithelial cells and ASM that we have not yet identified.

As PGAP3 is a GPI anchor-remodeling gene, we examined whether PGAP3 upregulated levels of GPI-anchored proteins. Of the 168 total GPI-APs and/or proteins that modify or bind to GPI-AP, that we identified on our UniProt search, there were five GPI-AP-encoding genes induced by PGAP3 in ASM-NA (SC) including genes that may influence cell adhesion (Mesothelin (MSLN)), as well as genes influencing vascular function (LY6/PLAUR Domain Containing 1 (LYPD1); Ephrin A5 (EFNA5)). These RT-qPCR studies are unable to determine whether levels of GPI-AP proteins (as opposed to mRNA) are modulated on the cell surface by increased expression of PGAP3. Insights into the ability of PGAP3 to modulate levels of cell surface GPI-AP are derived from studies of PGAP2 and PGAP3 mutant Chinese Hamster Ovary (CHO) cell lines, which have compared levels of selected cell surface GPI-AP (CD59, uPAR, and DAF) in wild type CHO cells deficient in PGAP2 and PGAP3 to levels in CHO cells stably transfected with both PGAP2 and PGAP3 [13]. The results of these studies demonstrated that stable transfection of PGAP2 and PGAP3 into cells deficient in both PGAP2 and PGAP3 increased levels of selected cell surface GPI-AP (CD59, uPAR, and DAF) [13]. Although these studies support a role for increased PGAP3 increasing GPI-AP proteins on the cell surface there are limitations in these studies in that the studies were performed in CHO cells deficient in both PGAP2 and PGAP3 and not only deficient in PGAP3 [13]. In addition, extending results from hamster ovary cell lines to primary human ASM requires further study.

Our study has limitations including using a plasmid with a promoter to express constitutive PGAP3 in ASM-NA in trying to recapitulate the same levels of PGAP3 constitutively expressed in ASM-A cells. We did perform initial pilot experiments in which we incubated different concentrations of PGAP3 plasmid with ASM-NA cells and quantitated the resultant level of PGAP3 mRNA by RT-qPCR. We chose the concentration of PGAP3 plasmid to use in our subsequent studies based on the concentration of PGAP3 plasmid that induced levels of PGAP3 mRNA in a range similar to that noted in our ASM-A cells. However, despite these precautions the levels of PGAP3 mRNA were higher in our plasmid transfected ASM-NA cells compared to ASM-A cells. Thus, these limitations need to be taken into account in the interpretation of the results.

In summary, this study made the novel observation that PGAP3 is expressed in ASM of asthmatics, and that ASM derived from the lungs of asthmatics express higher levels of PGAP3 compared to non-asthmatics. In addition, when ASM from non-asthmatics are transfected with PGAP3, the increased levels of PGAP3 increased ASM proliferation and contractility, and increased levels of genes previously linked to asthma including GATA3 and ALOX5. Overall, these studies suggest that increased PGAP3 expression in ASM plays a functional role in contributing to the pathogenesis of asthma.

## Supporting information

S1 Fig Gene set enrichment analysis of PGAP3-transfected ASM. (TIF)

S2 Fig Comparison of PGAP3-transfected ASM to UK BioBank asthma reference data set. (TIF)

S1 File Supplementary Table S1–S5. **Results of PGPA3-transfected ASM differential expression, comparisons to reference data sets, and bioinformatic analyses. Table S1.** Differentially Expressed Genes (p <  0.05) between PGAP3-transfected ASM vs. Expression Vector Controls after 24 Hours. **Table S2.** Differentially Expressed Genes (p <  0.05) between PGAP3-transfected ASM vs. Expression Vector Controls after 48 Hours. **Table S3.** Full List of PGAP3-Upregulated Genes in Normal Human Bronchial ASM Found in PGAP3-Regulated NHBE Reference Data Set. **Table S4.** Full List of PGAP3-Upregulated Genes in Normal Human Bronchial ASM Found in the UK BioBank Asthma Reference Data Set. **Table S5.** Network Topology Analysis highlighted seeds (log2 fold change ≥  2.0).
(XLSX)

## Abbreviations

AHR: Airway hyperreactivity
ALOX5: Arachidonate 5-Lipoxygenase
ASM: Airway smooth muscle
ASM-A (Ark): Airway smooth muscle-Asthma (Arkansas Children’s Research Institute Lung Cell Biology Laboratory)
ASM-NA (Ark): Airway smooth muscle-Non-Asthma (Arkansas Children’s Research Institute Lung Cell Biology Laboratory)
ASM-NA (SC): Airway smooth muscle-Non-Asthma (purchased from ScienCell)
FC: Fold change
GATA3: GATA Binding Protein 3
GSDMB: Gasdermin B
GWAS: Genome-wide association study
NHBE: Normal human bronchial epithelial cells
ORMDL3: ORMDL Sphingolipid Biosynthesis Regulator 3
PGAP3: Post-GPI Attachment to Proteins phospholipase 3
