# Supplementary figures and images for: PGAP3 is expressed at increased levels in asthmatic ASM and is associated with increased ASM proliferation, contractility and expression of GATA3 and ALOX5
Source: PLoS One. 2025 Mar 25;20(3):e0320427. doi: 10.1371/journal.pone.0320427 (PMC11936287; doi:10.1371/journal.pone.0320427)

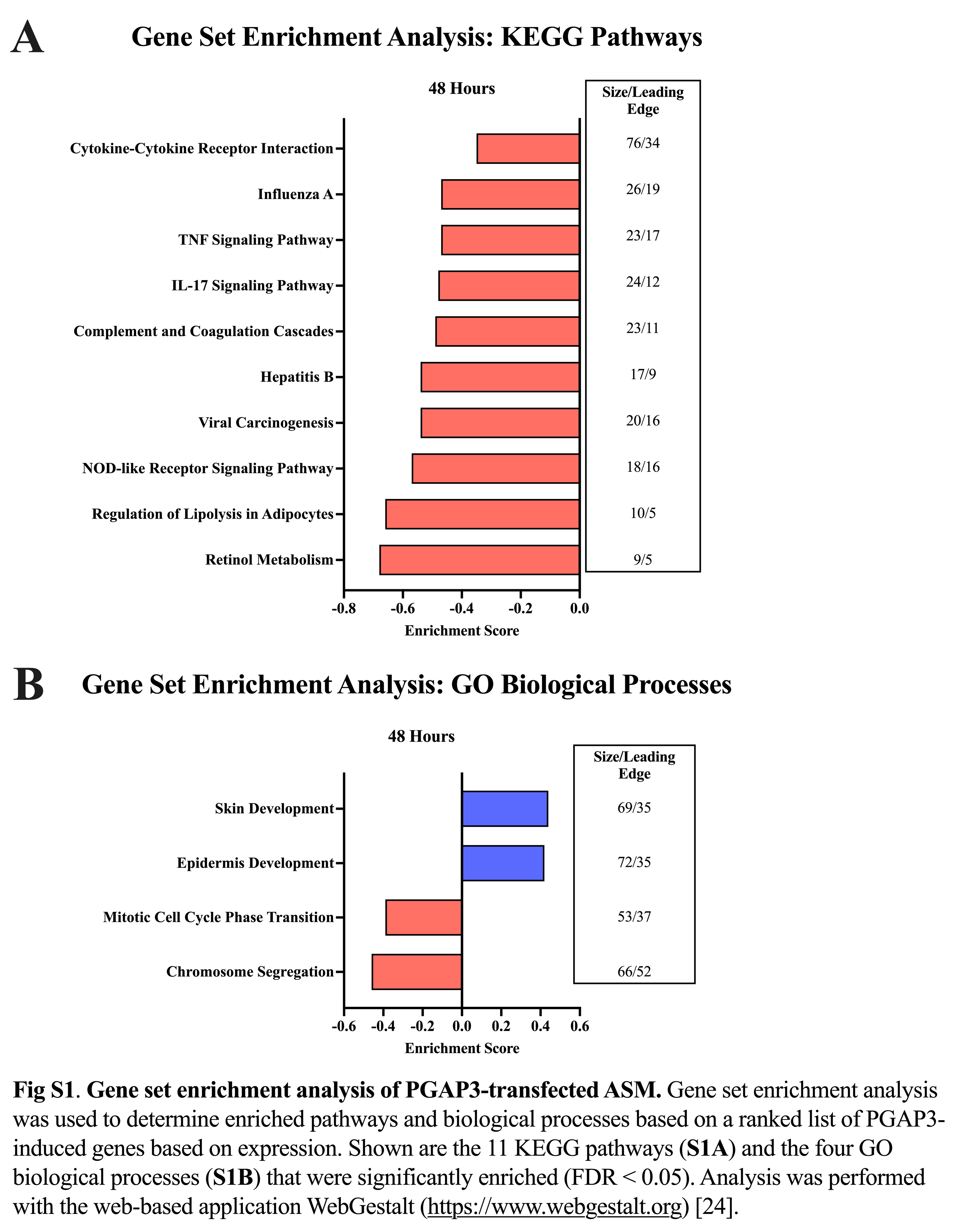

Supplement: S1 Fig — (TIF) [file pone.0320427.s001.tif]

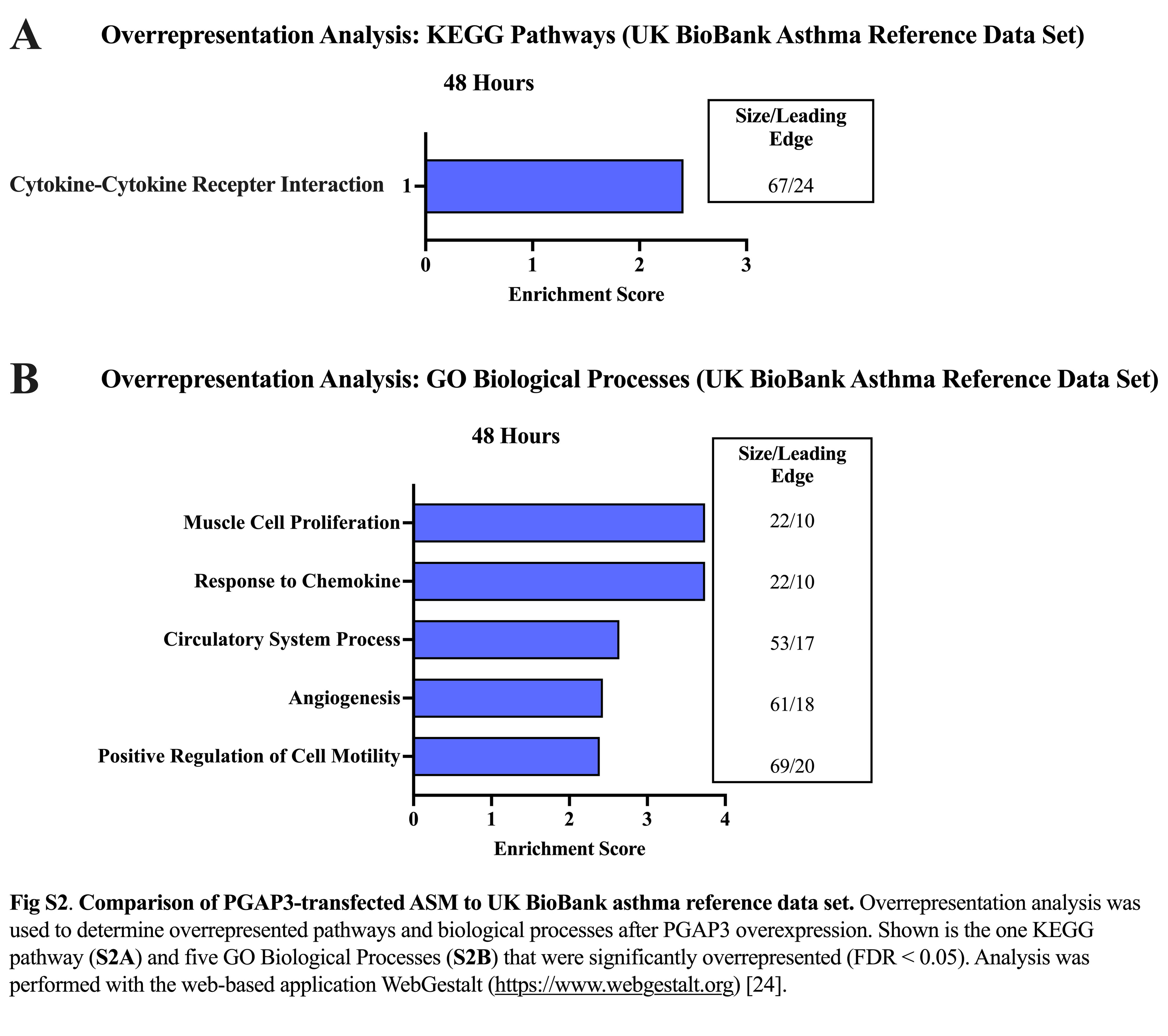

Supplement: S2 Fig — (TIF) [file pone.0320427.s002.tif]
